# A Longitudinal Study of Functional Outcomes in Patients with Limb Salvage Surgery for Soft Tissue Sarcoma

**DOI:** 10.1155/2018/6846275

**Published:** 2018-04-12

**Authors:** Eunsun Oh, Sung Wook Seo, Kwang Joon Han

**Affiliations:** ^1^Department of Radiology, Soonchunhyang University Seoul Hospital, 59 Daesagwan-ro, Youngsan-gu, Seoul 04401, Republic of Korea; ^2^Department of Radiology, Samsung Medical Center, Sungkyunkwan University School of Medicine, 81 Ilwon-Ro, Gangnam-gu, Seoul 06351, Republic of Korea; ^3^Department of Orthopedic Surgery, Samsung Medical Center, Sungkyunkwan University School of Medicine, 81 Ilwon-Ro, Gangnam-gu, Seoul 06351, Republic of Korea

## Abstract

**Background:**

Many studies have reported on the surgical outcomes of soft tissue sarcoma. However, there was no longitudinal cohort study. Because time is the most valuable factor for functional recovery, adjusting time value was the key for finding the causal relationship between other risk factors and postoperative function. Therefore, existing cross-sectional studies can neither fully explain the causal relationship between the risk factors and the functional score nor predict functional recovery. The aim of this study was to determine important predictive factors that affect postoperative functional outcomes and longitudinal changes in functional outcomes in patients who had undergone limb-sparing surgery (LSS) for soft tissue sarcoma (STS).

**Methods:**

Between January 2008 and December 2014, we retrospectively enrolled 150 patients who had undergone LSS for STS and had been assessed for postoperative functional outcomes with questionnaires. To evaluate functional outcomes, we used the Musculoskeletal Tumor Society (MSTS) score and Toronto Extremity Salvage Score (TESS). Multivariate generalized estimating equation (GEE) analysis was used to identify the predictive factors, including size, stage, and anatomic location of tumor, bone resection, flap reconstruction, age, and time after surgery. Each continuous variable such as age and time after surgery was explored for statistically significant cutoff points using the Wilcoxon rank sum test.

**Results:**

Functional scores significantly improved until the second year after surgery and plateaued for the rest of the 5-year period. Age (*p* < 0.0001), bone resection (*p*=0.0004), and time after surgery (*p* < 0.0001) were identified as significant predictive factors. The functional score was significantly higher in patients younger than 47 years old.

**Conclusions:**

Functional outcomes can improve until the second year after surgery. Patients who were older than 47 and underwent bone resection may have poor final functional outcomes.

## 1. Introduction

Soft tissue sarcomas (STSs) are rare malignant tumors, representing approximately 1% of all adult cancers [1]. Limb salvage surgery (LSS) is the preferred treatment for patients with STS rather than amputation [2, 3]. Multidisciplinary treatment that combines surgery with or without adjuvant radiation therapy has been widely applied with local control, but without any measurable decrease in disease-free survival and overall survival [4, 5]. However, surgical success can be assessed not only by oncological outcomes but also functional outcomes. Thus, functional outcomes after LSS are important to both surgeons and patients.

Many studies have reported predictors of functional outcomes for patients who have undergone LSS for STS. They evaluated functional outcomes at specific points, such as 6 months [6, 7] or 1 year [8] after surgery, or at an uncertain postoperative time point such as final follow-up [9]. These studies have limitations in not using longitudinal data with more than 2 time points and by not examining various predictors of postoperative functional outcomes for periods exceeding 5 years. With such reports, surgeons are not able to inform patients about the functional recovery period and final outcome after surgery.

Therefore, the purpose of our study was to determine important predictive factors that affect postoperative functional outcomes and longitudinal changes in functional outcomes in patients who had undergone LSS.

## 2. Materials and Methods

### 2.1. Study Design and Patients

The institutional review board approved this retrospective Health Insurance Portability and Accountability Act (HIPAA) compliant study (2015-10-176-001). Informed patient consent was waived. A retrospective cohort study of 212 patients who had undergone LSS for STS at our medical center between January 2008 and December 2014 was performed. All patients were assessed for postoperative functional outcomes with questionnaires. The functional outcomes were measured every 3 ± 1 months for 1 year postoperatively and every 6 ± 2 months from 2 to 5 years after surgery during their outpatient clinic visits. We included patients who had filled out questionnaires at least 2 times during a minimum follow-up period of 2 years. We excluded patients who already had functional disability at the operative site (*n*=24) or contralateral side (*n*=33) before surgery or accidental trauma irrelevant to the disease (*n*=5). Therefore, a total of 150 patients were included in this study.

### 2.2. Measurements

To evaluate functional outcomes, we used the Musculoskeletal Tumor Society (MSTS) score and Toronto Extremity Salvage Score (TESS). The MSTS score evaluates functional impairment after treatment and consists of 6 categories: pain, function, and emotional acceptance in the upper and lower extremities; supports, walking, and gait in the lower extremity; and hand positioning, dexterity, and lifting ability in the upper extremity [10]. It is measured by clinical physician through a standardized physical examination. Each category is rated on a scale of 0 to 5. The total score is calculated from the sum of each category and converted to a percentage value. The TESS evaluates performance of activities of daily living [11]. The upper and lower extremity versions of the TESS have 29-item and 30-item questionnaires, respectively. Each item is rated on a scale of 0 to 5. The point score is obtained, and the percentage is calculated. We used the translation and cross-cultural adaptation of the Korean version of TESS [12]. Kim et al. [12] demonstrated that two bilingual translators translated the original version of the TESS questionnaire into Korean then translated back into English. The Korean version of TESS was reviewed by a committee to develop the consensus. The Korean version of TESS was administered to 126 patients to examine its comprehensibility, reliability, and validity.

Several known predictors of functional outcome include patient age, size and grade of tumor, irradiation, and presence of bone and motor nerve resection [6]. Based on these, we defined several potential predictive factors that could have an influence on functional outcomes: size, stage and anatomic location of tumor, bone resection (no/yes), flap reconstruction (no/yes), postoperative radiation (no/yes), patient age, and time after surgery. The size of tumor was based on maximum diameter in centimeters. To assign a stage to a patient with STS, we adopted the 7th edition of the *American Joint Committee on Cancer (AJCC) Cancer Staging Manual* [13], which is widely accepted as an important surgical consideration. We divided tumor stage into 3 groups: group 1, stages I and IIA; group 2, stages IIB and III; and group 3, stage IV. The anatomic location of tumor was classified into upper extremity, lower extremity, and trunk.

### 2.3. Statistical Analysis

All statistical analyses were performed using statistical software (SAS version 9.4, SAS Institute, Cary, NC, USA; R version 3.1.0, R Development Core Team, Vienna, Austria). A box plot was applied to explore the statistical distribution of functional scores in the MSTS and TESS. Multivariate generalized estimating equation (GEE) analysis was used to identify the predictive factors that could affect the functional outcomes. Longitudinal data were repeated, and measures were obtained from the same subjects. High correlation within the same patient pool indicated that longitudinal relationships could not be analyzed by common regression methods, which presumed the independence of data [14, 15]. Therefore, the GEE analysis was the appropriate statistical methodology. For the significant predictors of functional outcome by multivariate GEE analysis, the optimal cutoff was chosen as the point with the most significant Wilcoxon rank sum test *p* value for all possible cutoff points. For all statistical analysis, differences were considered to be significant if the *p* value was less than 0.05.

## 3. Results

**Figure 1:**
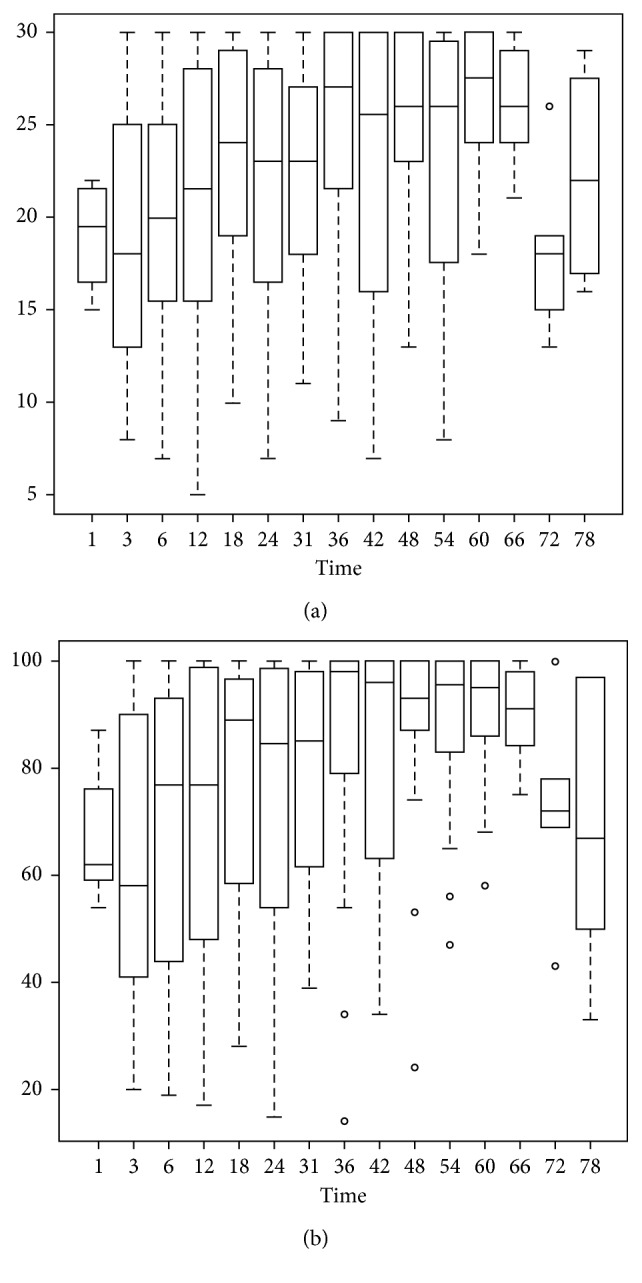
Box plot of overall (a) MSTS and (b) TESS during the entire follow-up period.

**Figure 2:**
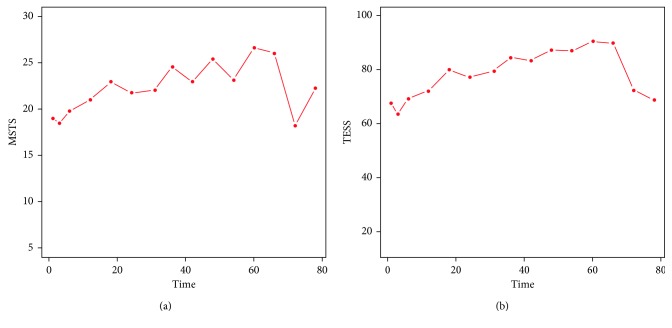
Mean plot of overall (a) MSTS and (b) TESS during the entire follow-up period.

The characteristics of all 150 patients, comprising 81 men (54%) and 69 women (46%), are listed in Table 1. The mean age at the time of surgery was 47 years (range 10–90). The distribution graphics of functional scores in the MSTS and TESS are shown in Figures 1 and 2. The functional scores in the MSTS and TESS significantly improved until the second year after surgery and then remained stable for the rest of the 5-year period. The optimal cutoff was chosen as 24 months, based on the most significant Wilcoxon rank sum test *p* value for all possible cutoff points.

Table 2 shows the results of multivariate GEE analysis of the effective predictive factors of functional outcomes. Among all factors, time after surgery was significantly related to both MSTS (*p*=0.001) and TESS (*p*=0.0004). There were significant differences in functional scores by age in the MSTS and TESS (*p* < 0.0001). Older patients scored lower than younger patients in functional outcomes. The optimal cutoff was chosen as 47 years old, based on the most significant Wilcoxon rank sum test *p* value for all possible cutoff points.

There were also significant differences in functional scores according to bone resection in the MSTS and TESS (*p* < 0.0001). Patients who had undergone bone resection surgery scored lower in both MSTS (6.1%) and TESS (19.3%) than those who had not undergone bone resection. Thus, even when postoperative time is considered, patients with bone resection had a 20% lower functional recovery.

## 4. Discussion

This study is the first to determine predictive factors associated with postoperative functional outcomes through a longitudinal study using the GEE approach to explain trends over time. In our study, we found that age, bone resection, and time after surgery were significant predictive factors of functional outcomes. Moreover, functional outcomes in the MSTS and TESS over a 5-year follow-up tended to improve until the second year.

Our study demonstrated that functional outcomes in the MSTS and TESS improved until the second year after surgery, and plateaued for the rest of the 5-year period. Previous studies used a minimum follow-up period of 6 months [6, 7] or 1 year [8, 16] for evaluation of functional outcomes, at which point functional scores are known to plateau. However, in our study, 24 months was the significant cutoff point for functional outcomes; functional scores before 24 months were not appropriate to evaluate the final functional outcome after surgery. Time after surgery was an independent predictive factor of functional outcome in our study. Therefore, time after surgery could be a confounding factor when comparing functional scores that were measured at different time points.

This study indicated that age was another independent predictive factor of functional outcomes. Older patients showed less improvement than younger patients. This may be because older patients tended to have more comorbidity. Additionally, there was a significant difference at a cutoff point of age 47.

Bone resection was another independent predictive factor of functional outcomes. Davis et al. [6] showed that bone resection was significantly related to increased disability on the MSTS using univariate and multivariate analysis and the TESS using only univariate analysis. In our study, patients who had undergone bone resection showed poorer functional outcomes on the MSTS and TESS than those without resection, regardless of time after surgery. This suggested that wide bone resection including muscle resulted in significant functional disability. Based on these data, surgeons can expect a poor functional outcome when performing bone resection, and further study is needed to solve this problem.

The size and stage of tumor indicated the degree of impairment in adjacent structures as well as the extent of tumor removal, and could have an effect on functional outcomes after surgery. In contrast with our expectation, the size and stage of tumor did not have a significant effect on the MSTS and TESS. The reason is that patients with advanced stage and large tumor size died during the follow-up and the patients were not available for assessment, we suggested. Therefore, further prospective study and randomized controlled trials should be performed to clarify the significance of size and stage of tumor.

Our study has several limitations. First, the number of patients followed for more than 5 years declined rapidly, and it was difficult to calculate an accurate value based on more than 5-year follow-up results. Second, we did not evaluate the preoperative functional score, local tumor recurrence, metastasis, surgical complications, indication and extent of bone resection, and type and methods of flap reconstruction, which can affect subsequent treatment and potential functional outcomes. It was a fundamental limitation of our study, and we will need more research in the future. Third, in the case of upper extremity tumors, we did not divide their locations into dominant arm and non-dominant arm. This may have had an impact on the potential functional outcomes. Fourth, the number of low grade tumors was much higher than that of high grade tumors, resulting in a relatively high level of functional outcome. Finally, MSTS and TESS were designed primarily as a simple way to measure the function of a single extremity. These systems had potential limitations in understanding the overall quality of life. Therefore, more research is needed to measure a broader understanding of the patients' overall recovery.

In conclusion, functional outcomes in patients who undergo LSS for STS can improve until the second year after surgery. The clinician can reassure patients that functional outcome improves gradually, and that final functional outcome will be better than early postoperative outcome. In addition, there is a significant difference in functional outcome between patients younger and older than 47 years of age. Patients who underwent bone resection may have a poor final outcome.

## Figures and Tables

**Table 1:** Clinical and demographic characteristics of patients (*n*=150).

Characteristic	Number	Percentage (%)
Patient attributes		
Age^a^ (years)	47.04 ± 17.19	
Gender		
Male	81	54
Female	69	46
Tumor attributes		
Size^a^	6.81 ± 5.79	
Stage group		
Group 1	78	52
Group 2	67	44.67
Group 3	5	3.33
Anatomic location		
Upper extremity	38	25.33
Lower extremity	102	68
Trunk	10	6.67
Surgery attributes		
Bone resection		
No	117	78
Yes	33	22
Flap reconstruction		
No	102	68
Yes	48	32
Postoperative radiation		
No	102	68
Yes	48	32

^a^Mean ± standard deviation.

**Table 2:** Multivariate generalized estimating equation (GEE) analysis of predictors of functional outcome in MSTS and TESS.

Variables^a^		MSTS			TESS		
		Estimate	Standard error	*p* value (95% CI)	Estimate	Standard error	*p* value (95% CI)
Intercept		29.39	1.84	<0.0001 (25.78, 33.00)	101.81	7.20	<0.0001 (87.68, 115.94)
Time		0.05	0.01	0.001 (0.02, 0.08)	0.21	0.06	0.0004 (0.09, 0.33)
Age		−0.15	0.02	<0.0001 (−0.21, −0.10)	−0.54	0.10	<0.0001 (−0.75, −0.33)
Size		−0.01	0.08	0.86 (−0.18, 0.15)	−0.13	0.29	0.64 (−0.72, 0.45)
Stage group							
Group 3	3	−2.86	1.83	0.11 (−6.46, 0.73)	−10.90	6.53	0.09 (−23.72, 1.90)
Group 2	2	−0.76	0.93	0.41 (−2.60, 1.06)	−4.50	3.40	0.18 (−11.18, 2.17)
Group 1	1	0	0		0	0	
Anatomic location							
Trunk	3	3.14	1.85	0.08 (−0.48, 6.77)	16.90	8.11	0.03 (1.00, 32.79)
Lower extremity	2	0.59	1.07	0.57 (−1.51, 2.71)	0.89	4.34	0.83 (−7.62, 9.41)
Upper extremity	1	0	0		0	0	
Bone resection		−6.13	1.24	<0.0001 (−8.57, −3.68)	−19.28	4.38	<0.0001 (−27.89, −10.68)
Flap reconstruction		−0.11	1.00	0.91 (−2.08, 1.86)	2.34	3.94	0.55 (−5.38, 10.07)
Postoperative radiation		−1.14	1.26	0.36 (−3.62, 1.32)	−6.07	4.65	0.19 (−15.18, 3.04)

^a^Bone resection: 0 = no, 1 = yes; flap reconstruction: 0 = yes, 1 = no; CI: confidence interval.
